# Tocotrienols and Whey Protein Isolates Substantially Increase Exercise Endurance Capacity in Diet -Induced Obese Male Sprague-Dawley Rats

**DOI:** 10.1371/journal.pone.0152562

**Published:** 2016-04-08

**Authors:** Andrew C. Betik, Jay Aguila, Glenn K. McConell, Andrew J. McAinch, Michael L. Mathai

**Affiliations:** 1 Centre for Chronic Disease, College of Health and Biomedicine, Victoria University, Melbourne, Victoria, Australia; 2 Institute of Sport, Exercise and Active Living (ISEAL), Victoria University, Melbourne, Victoria, Australia; Monash University, AUSTRALIA

## Abstract

**Background and Aims:**

Obesity and impairments in metabolic health are associated with reductions in exercise capacity. Both whey protein isolates (WPIs) and vitamin E tocotrienols (TCTs) exert favorable effects on obesity-related metabolic parameters. This research sought to determine whether these supplements improved exercise capacity and increased glucose tolerance in diet-induced obese rats.

**Methods:**

Six week old male rats (n = 35) weighing 187 ± 32g were allocated to either: Control (n = 9), TCT (n = 9), WPI (n = 8) or TCT + WPI (n = 9) and placed on a high-fat diet (40% of energy from fat) for 10 weeks. Animals received 50mg/kg body weight and 8% of total energy intake per day of TCTs and/or WPIs respectively. Food intake, body composition, glucose tolerance, insulin sensitivity, exercise capacity, skeletal muscle glycogen content and oxidative enzyme activity were determined.

**Results:**

Both TCT and WPI groups ran >50% longer (2271 ± 185m and 2195 ± 265m respectively) than the Control group (1428 ± 139m) during the run to exhaustion test (P<0.05), TCT + WPI did not further improve exercise endurance (2068 ± 104m). WPIs increased the maximum *in vitro* activity of beta-hydroxyacyl-CoA in the soleus muscle (P<0.05 vs. Control) but not in the plantaris. Citrate synthase activity was not different between groups. Neither supplement had any effect on weight gain, adiposity, glucose tolerance or insulin sensitivity.

**Conclusion:**

Ten weeks of both TCTs and WPIs increased exercise endurance by 50% in sedentary, diet-induced obese rats. These positive effects of TCTs and WPIs were independent of body weight, adiposity or glucose tolerance.

## Introduction

Obesity and its related comorbidities are major public health threats that are escalating rapidly. Physical activity, along with calorie restriction, is regarded as one of the first line strategies to prevent or treat obesity and its associated comorbidities [1]. Exercise tolerance, while a strong predictor of morbidity and mortality risk [2–4], is reduced in obese and/or metabolically impaired animals and humans [5–7]. Thus long-term maintenance of increased physical activity levels in obese individuals may be difficult to achieve [8].

There is evidence that obesity is associated with reduced skeletal muscle oxidative capacity [9–12]. The reduced oxidative capacity has been postulated to provoke perturbations in insulin signalling, leading to the eventual development of insulin resistance and type 2 diabetes [10, 13]. Notably, these impairments in skeletal muscle oxidative metabolism are known to contribute to the reduced exercise tolerance displayed in obese humans and animals [7, 14, 15]. Exercise is a proven intervention that can robustly improve muscle oxidative capacity, insulin sensitivity, and reduce body adiposity and as such, it can help combat these metabolic diseases [16].

Whey protein isolates (WPIs), through their high content of branched-chain amino acids and proline-rich peptides eliciting angiotensin converting enzyme inhibitory effects, have also been postulated as a potential treatment to combat obesity and its comorbidities [17]. Supplementation with WPI has been shown to improve blood pressure, glucose tolerance, insulin sensitivity, adipose mass and blood lipid profiles in populations with metabolic syndrome [18]. Leucine, a major component of whey protein, has also been shown to increase fatty acid oxidation in muscle cells and decrease fatty acid synthase expression in adipocytes [19] suggesting an anti-obesity role, although the concentrations of leucine used in these cell culture experiments is substantially higher than that seen in the blood. One recent study showed that short-term supplementation with WPIs increased the gene expression of peroxisome proliferator-activated receptor gamma coactivator 1 alpha (PGC-1α) [20], a transcription factor considered to be the master regulator of mitochondrial biogenesis [21, 22]. This finding supports earlier mechanistic studies which show that leucine increases mitochondrial mass and oxygen consumption by inducing mitochondrial biogenesis in muscle cells [23, 24]. While a large body of evidence supports substantial benefits of WPIs on protein synthesis post-resistance exercise [25, 26], the effect of WPIs supplementation on endurance exercise capacity has yet to be investigated.

Tocotrienols (TCTs) and tocopherols are members of the Vitamin E family and are naturally occurring lipid-soluble nutrients that consist of four corresponding homologues (α-, β-, γ- and δ-) [27]. Unlike tocopherols, TCTs have been shown to elicit strong antihypercholesterolemic [28], antihypertensive [29], and anti-diabetic effects [30]. The majority of studies on Vitamin E have investigated tocopherol due to its strong antioxidant properties, however there is some evidence that TCT can also improve metabolic parameters such as serum lipids and cholesterol in people with diabetes [31]. While the mechanism is unknown, TCT have been shown in cells to activate peroxisome proliferator-activated receptors (PPAR’s) [32], which are transcription factors related to genes involved in fatty acid oxidation, as well as activating PGC-1α. Furthermore, a recent study has shown that TCTs enhance exercise tolerance by over 2-fold in rats by attenuating muscle and liver glycogen use whilst reducing the increase in plasma lactic acid levels during exercise [33]. In that study, Wistar rats fed two doses (25 and 50mg/kg body weight) of TCT remarkably took 98% and 145% longer, respectively, to swim to exhaustion than normal chow fed rats [33]. The higher muscle and liver glycogen and lower blood lactate at the end of exercise in the TCT groups suggest that the TCTs enhanced fat oxidation and thus reduced carbohydrate oxidation [33]. Increased fat oxidation may be due to the activation of the PPARs and mitochondrial biogenesis which could also explain the increased exercise capacity. The effects of TCT supplementation on exercise capacity in obesity is however unknown.

This study aimed to investigate the effects of dietary supplementation with TCTs and WPIs, in isolation and in combination, on metabolic parameters at rest and in response to exercise, in rodents fed a high fat diet (HFD) for 10 weeks to induce an obese state. It was hypothesized that TCTs and WPIs during the HFD would attenuate the increase in adiposity from the HFD as well as increase glucose tolerance compared with high fat diet alone. Secondly, it was hypothesized that WPIs and TCTs would improve exercise endurance capacity, as determined by a submaximal intensity prolonged run to exhaustion on a motorised rodent treadmill. Furthermore, we hypothesized that the TCT groups would have higher post-exercise glycogen content in the liver and skeletal muscles, as observed previously [33], along with lower blood lactate levels, indicative of higher rates of fatty acid oxidation and lower carbohydrate oxidation during the exercise bout.

## Methods

### Experimental Groups

All procedures performed involving animals were in accordance with the Australian Code of Practice for the Care and Use of Animals for Scientific Purposes (2013, 8th Edition) and were approved by Victoria University and Howard Florey Institute Animal Ethics Committee’s (AEC 12-025-FNI).

**Fig 1:**
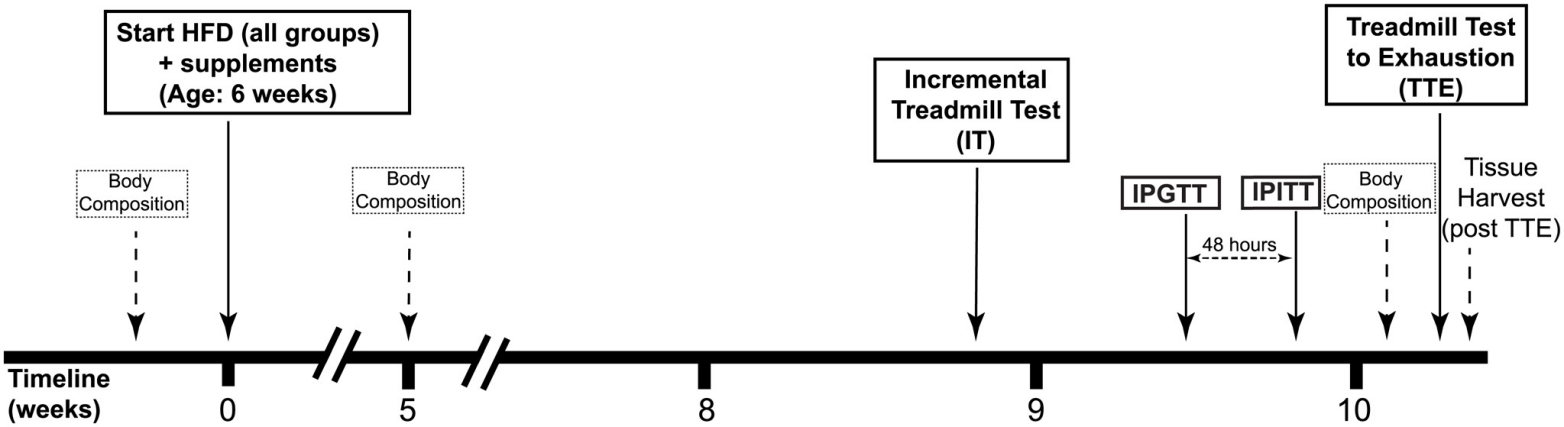
Experimental timeline outlining the tests with respect to the 10 weeks of high fat diet and supplementation. HFD = High fat diet; IPGTT—Intraperitoneal glucose tolerance test; IPITT = Intraperitoneal insulin tolerance test; IT—Incremental treadmill test; TTE = Treadmill test to exhaustion; IT occurred near the end of Week 8; IPGTT and IPITT occurred near the end of Week 9 with 48 hours between each test; TTE occurred near the end of Week 10.

35 male Sprague-Dawley rats (age: 6 weeks, body weight: 187±32g) were randomly assigned to one of the four treatment groups: a high fat fed control (Control), consisting of a high-fat/high-sucrose diet (HFD) (40% of energy from fat, 35% from sucrose) (purchased from Specialty Feeds: Glen Forrest, WA, Australia; catalogue # SF00-219); TCT, consisting of a HFD plus TCTs (Tocomin^®^, 50mg/kg body weight per day, Carotech BHD, Perak, Malaysia); WPI, consisting of a HFD plus WPIs (8% of total energy intake; containing >90% WPIs, Murray Goulburn Nutritionals, Victoria, Australia) and TCT + WPI, consisting of a HFD plus TCTs (50mg/kg body weight) and WPIs (8% of total energy intake). HFD has been shown to induce insulin resistance [34–36] and obesity compared to normal chow diet [37, 38]. Throughout the 10-week intervention, rats were maintained on a 12 hour light/dark cycle and kept at 22–24°C at approximately 40% relative humidity with *ad libitum* access to food and water. Food intake and body weight were measured daily and body composition data from each rat was obtained before the supplementation and weeks 5 and 10 (Fig 1) using an EchoMRI^™^ Whole Body Composition Analyzer (EchoMRI-900, Houston, TX, USA).

### Intraperitoneal Glucose and Insulin Tolerance Tests

An intraperitoneal glucose tolerance test (IPGTT) was performed in each rat at the end of week 9, as previously described [38]. To summarize, following an overnight fast, rats were administered 2g/kg body weight of glucose solution (Sigma G-7528) via a 3ml syringe injection into the peritoneal cavity. Prior to the injection of glucose, rats had their fasting blood glucose levels determined via tail snip and subsequent use of glucose strips (FreeStyle Optium Blood Glucose Test Strips, Abbott Diabetes Care, Doncaster, VIC, Australia) that were automatically read with a hand-held glucometer (Roche Diagnostics, Castle Hill, NSW, Australia). Thereafter, tail snip blood glucose levels were recorded 15, 30, 60, 90 and 120 minutes after the administration of glucose.

An intraperitoneal insulin tolerance test (IPITT) was performed 48 h after the IPGTT as previously described [38]. Briefly, food was removed 2 hours prior to the test and then each rat received 0.75 U/kg of body weight of insulin (purchased from Elanco, Eli Lilly, Sydney, Australia) through injection into the peritoneal cavity using a 1ml syringe. Rats had their blood glucose levels determined prior to the insulin injection by tail snip. Thereafter, blood glucose levels were recorded 15, 30, 60, 90 and 120 minutes after the administration of insulin by tail snip.

### Exercise Protocol

All animals were familiarized to a motor-driven treadmill (Columbus Instruments, Columbus, OH, USA) by running for 10 minutes, twice per week between week 4 and week 8 at a speed of 15.7 m/min and 8° incline, as adapted from Lee, Bruce [39]. At the end of week 8, all animals performed an incremental test (IT) to exhaustion to determine maximum running capacity. This test entailed commencing the treadmill speed at 10 m/min and increasing by 2.5 m/min every 2 min until exhaustion, a protocol that has been performed previously for determining maximal aerobic running capacity in rats [40, 41]. Exhaustion was determined when the rat stopped running and had to be replaced onto the treadmill 3 successive times despite encouragement using a tail brush, tail pinching or compressed air on the tail [40, 42]. At the end of week 10, all rats performed a time to exhaustion (TTE) test at a treadmill speed set to 65% of their individually determined maximal velocity reached during the IT that was performed two weeks prior. There was no difference in body weight between any of the groups at the time of the IT (Week 8) or the TTE (Week 10), however all groups gained approximately 7–8% of body weight between the two tests. As such, it would be expected that the intensity during the TTE was greater than 65% of their maximal capacity at this time point. After 90 minutes of running at this set speed, the speed of the treadmill was increased by 2 m/min every 15 minutes until the animal had reached exhaustion using the same criteria as the IT. To accommodate for potential investigator differences in deeming exhaustion and to minimize the potential for bias, the same experimenter judged the exhaustion of all rats in both the IT and TTE, and this experimenter was blinded to all groups throughout all exercise performance tests, and the rats performed the tests in a randomized order.

### Tissue and blood collection

Immediately following the TTE, rats were anaesthetised by isoflurane and euthanized by cardiac puncture (within 5 min from the end of exercise) and blood was collected from the left ventricle into a heparin-coated 10ml tube (BD Vacutainer^®^, Plymouth, UK). The sample tube was immediately cooled on ice and centrifuged (Sorvall^™^ RT7 Benchtop Centrifuge) within 20 minutes at 900 g for 15 minutes at 4°C, after which plasma was separated and stored at -80°C. Both soleus (primarily type I slow fibres) and plantaris (>90% type II fast fibres) [43] muscles were quickly dissected out and immediately frozen in liquid nitrogen. Following this, the left cardiac ventricle and epididymal and brown fat pads were removed and weighed.

### Post-exercise plasma lactate and glucose, and skeletal muscle enzymes and glycogen analysis

Post-exercise plasma glucose and lactate were determined by using an automated glucose analyser machine (YSI 2300 STAT Plus Glucose & Lactate Analyzer, YSI Incorporated, USA). Whole frozen muscles were pulverised using a mortar and pestle (pre-submerged in liquid nitrogen) to obtain a representative sample of muscle fibres from all muscle samples [42], and the oxidative capacity of the soleus and plantaris muscles were determined through measuring the activity of citrate synthase (CS) and beta-hydroxyacyl-CoA (β-HAD) as previously described [44, 45]. Enzyme activities were expressed relative to wet weight. Muscle glycogen content was determined via an enzymatic assay adapted for fluorometry and modified for a microplate reader [20, 46]. Powdered tissue samples were homogenized in 2M hydrochloric acid (1mg: 50μL) for 2 hours (100°C) and then neutralised with 0.66M NaOH (1mg: 150μL). 5 μL of sample was added in triplicate to a 96 well plate, followed by 250 μL reagent cocktail (1.0 M Tris Buffer pH 8.8, 1.0M MgCL_2_, 0.5 M DTT, 0.3M ATP, NADP^+^, 5mg/mL glucose-6-phosphate dehydrogenase) and an initial reading made using a fluorometer (Excitation 355nm, Emission: 450nm, Fluoroskan Ascent FL, Thermo Fisher Scientific, Waltham, MA, USA). 8 μL of diluted hexokinase (ratio 25μL: 1mL reagent cocktail) was then added to each well to commence the reaction which continued in the dark at room temperature for 1 hour (endpoint reaction) after which another reading on the fluorometer was taken.

### Statistical Analysis

All data are expressed as mean ± standard error of the mean (SEM). A one-way analysis of variance (ANOVA) was performed to assess total accumulated food and energy intake, muscle enzyme activities, muscle glycogen content, tissue weights, post-exercise serum glucose and lactate levels, and physical performance tests (IT and TTE). Changes in body composition and body weight were evaluated by using a repeated-measures ANOVA design. The Area under the curve for the IPGTT and IPITT was determined using GraphPad Prism 5 software (GraphPad Software Inc, La Jolla, CA, USA), and this data was subsequently analysed with a one-way ANOVA. Differences were considered statistically significant if the P value was less than 0.05. All statistical calculations were conducted using SPSS 19.0 (SPSS Inc., Chicago, IL, USA).

## Results

### Total Energy Intake, Body Weight and Body Composition

**Fig 2:**
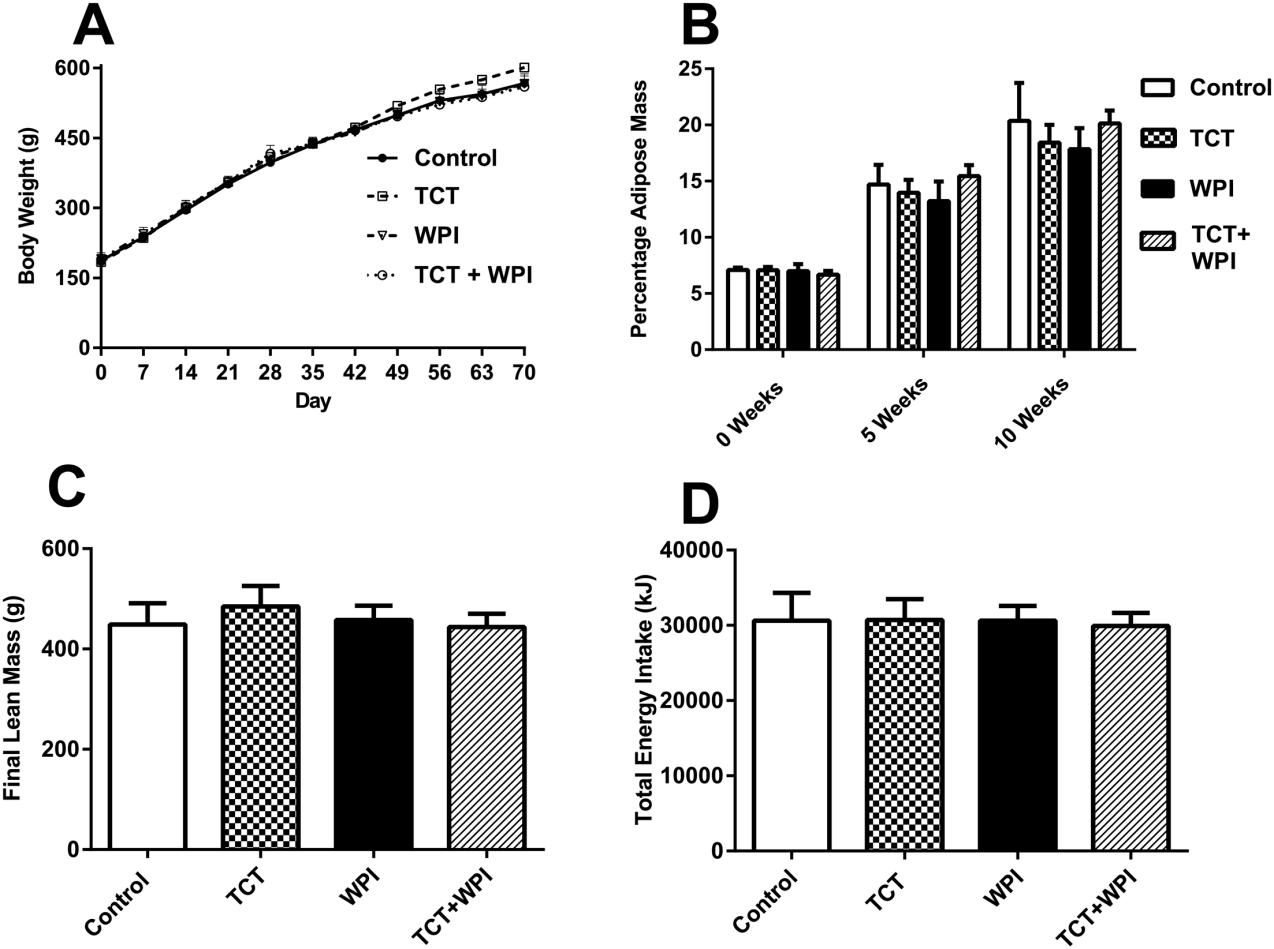
Effect of TCT- and/or WPI- supplementation on body weight (A), percent body fat (B), lean mass (C) and total energy intake (D) throughout the 10 week intervention study. A) Body weight of all experimental groups throughout the 10-week experimental study. B) Percentage body fat of each of the four groups at the start of experiment, 5 weeks and 10 weeks of supplementation. C) Final lean mass at the end of the 10 weeks supplementation period. D) Calculated absolute total energy intake of each four treatment groups throughout the 10-week intervention study. All data are presented as mean ± SEM (n = 8−9/group) and there were no significant differences between groups. A main effect for time was evident for body mass and adiposity (p<0.05). Control = HFD alone; TCT = HFD+tocotrienol; WPI = HFD+whey protein isolate; TCT+WPI = HFD+tocotrienol+whey protein isolates.

The initial starting weights (186.8 ± 10.2g, 184.3 ± 11.3g, 190.6 ± 13.3g and 186.6 ± 10.7g) were similar for the Control, TCT, WPI and TCT+WPI groups, respectively. Accumulation of body weight and final body weight following the 10-week intervention were not different between the groups (Control, 570.7 ± 19.9g; TCT 604.2 ± 21.4g; WPI, 567.1 ± 17g; TCT+WPI, 565.3 ± 13.4g) (Fig 2A). All groups started before diet supplementation with comparable body fat levels (approximately 7%) and there was no difference in adiposity at week 5 or week 10 between the groups (Fig 2B). Likewise, all groups had similar lean body mass levels at week 10 (Fig 2C). Total energy intake throughout the 10-week intervention study was comparable across all the cohorts (Fig 2D). The level of adiposity (18–20%) in the HFD groups is similar to studies from our group [37, 38] and others [47, 48] that have shown the HFD results in higher adiposity and body mass compared with a standard chow diet group. Jenkin et al. [38] reported body masses of 563g vs 622g and adiposity of 9.5% vs 17% in chow fed and 9 weeks HFD groups respectively, when fed identical diets to the current study. Similarly, after 12 weeks of HFD, Cornall et al. reported [37] body masses of 548g vs 635g and adiposity of 13% vs 24% in 18 week old chow fed and HFD groups respectively.

### Tissue Weights

No differences in the mass of the epididymal and intrascapular fat pads and cardiac left ventricle were detected between the cohorts (Table 1).

**Table 1:** Effect of TCT- and/or WPI- supplementation on Epididymal (white fat) and Intrascapular (brown fat) adipose tissues and cardiac left ventricle mass in rats.

Group	Brown Fat (g)	Epididymal Fat (g)	Cardiac Left Ventricle (g)
**Control**	0.73 ± 0.08	21.9 ± 3.2	1.17 ± 0.04
**TCT**	0.78 ± 0.05	27.6 ± 3.2	1.24 ± 0.04
**WPI**	0.72 ± 0.04	24.7 ± 2.6	1.19 ± 0.05
**TCT + WPI**	0.79 ± 0.07	19.7 ± 2.3	1.23 ± 0.05

Data presented as mean ± SEM (n = 7−9/group). Control = HFD alone; TCT = HFD+tocotrienol; WPI = HFD+whey protein isolate; TCT+WPI = HFD+tocotrienol+whey protein isolates. No significant differences exist for any of the measures.

### Assessment of Glucose Homeostasis via IPGTT and IPITT

**Fig 3:**
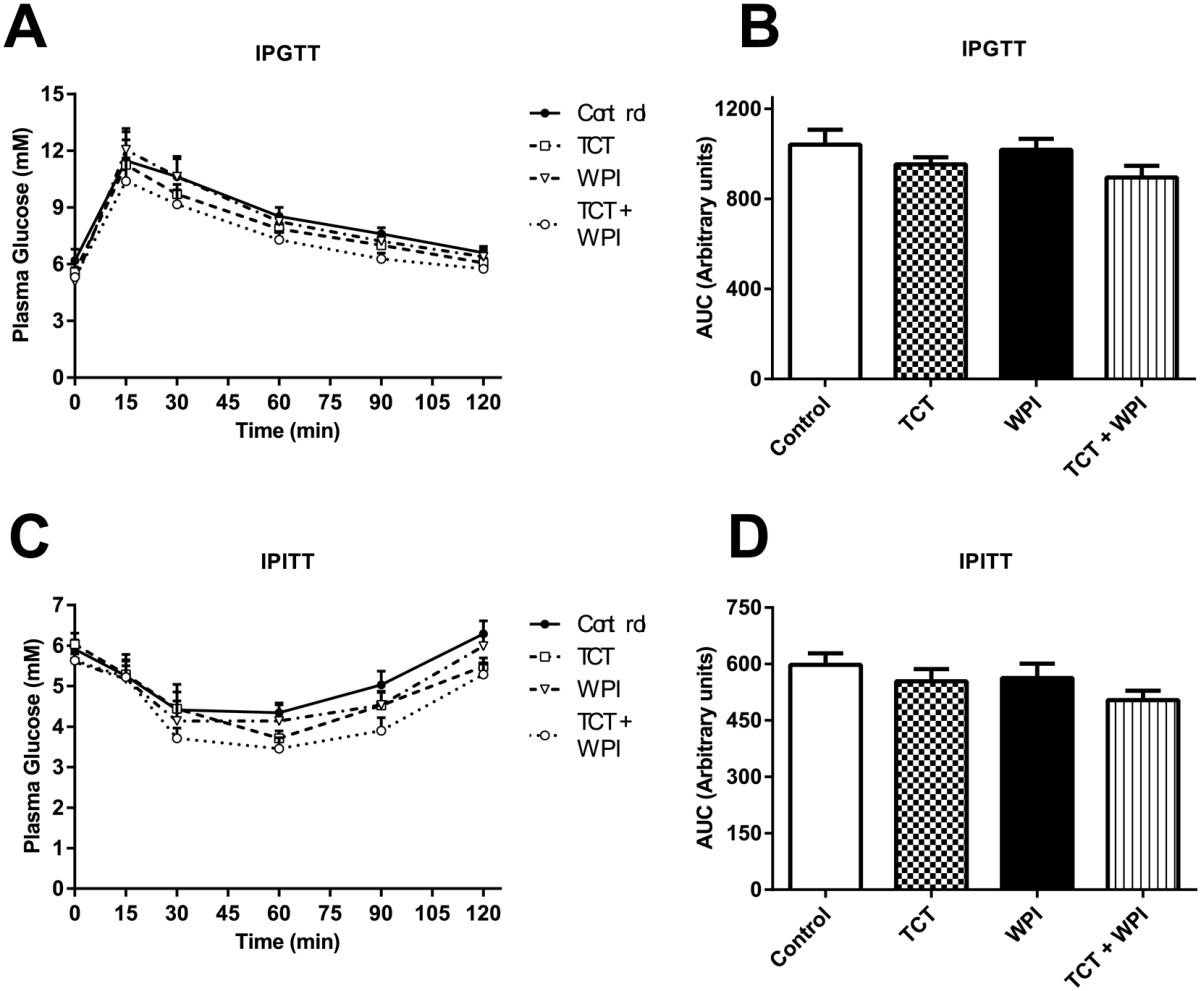
Plasma glucose and area under the curve (AUC) during the IPGTT and IPITT. IPGTT performed on rats at the end of the 9^th^ week along with corresponding AUC data obtained (A and B, respectively). IPITT performed on rats 2 days after the IPGTT along with corresponding AUC data obtained (C and D, respectively). Data are presented as mean ± SEM (n = 8−9/group). No significant differences were detected between groups for either test, although there was a tendency for TCT+WPI group to be lower (p = 0.1) for the IPITT. Control = HFD alone; TCT = HFD+tocotrienol; WPI = HFD+whey protein isolate; TCT+WPI = HFD+tocotrienol+whey protein isolates.

During the IPGTT, there was no between groups difference in blood glucose concentrations (Fig 3A). Similarly, no effect on blood glucose concentrations between the groups was observed during the IPITT (Fig 3C). Consequently, the areas under the curves for the four groups for both the IPGTT and IPITT (p = 0.1) were not statistically different from one another (Fig 3B and 3D).

### Physical Performance Tests

**Fig 4:**
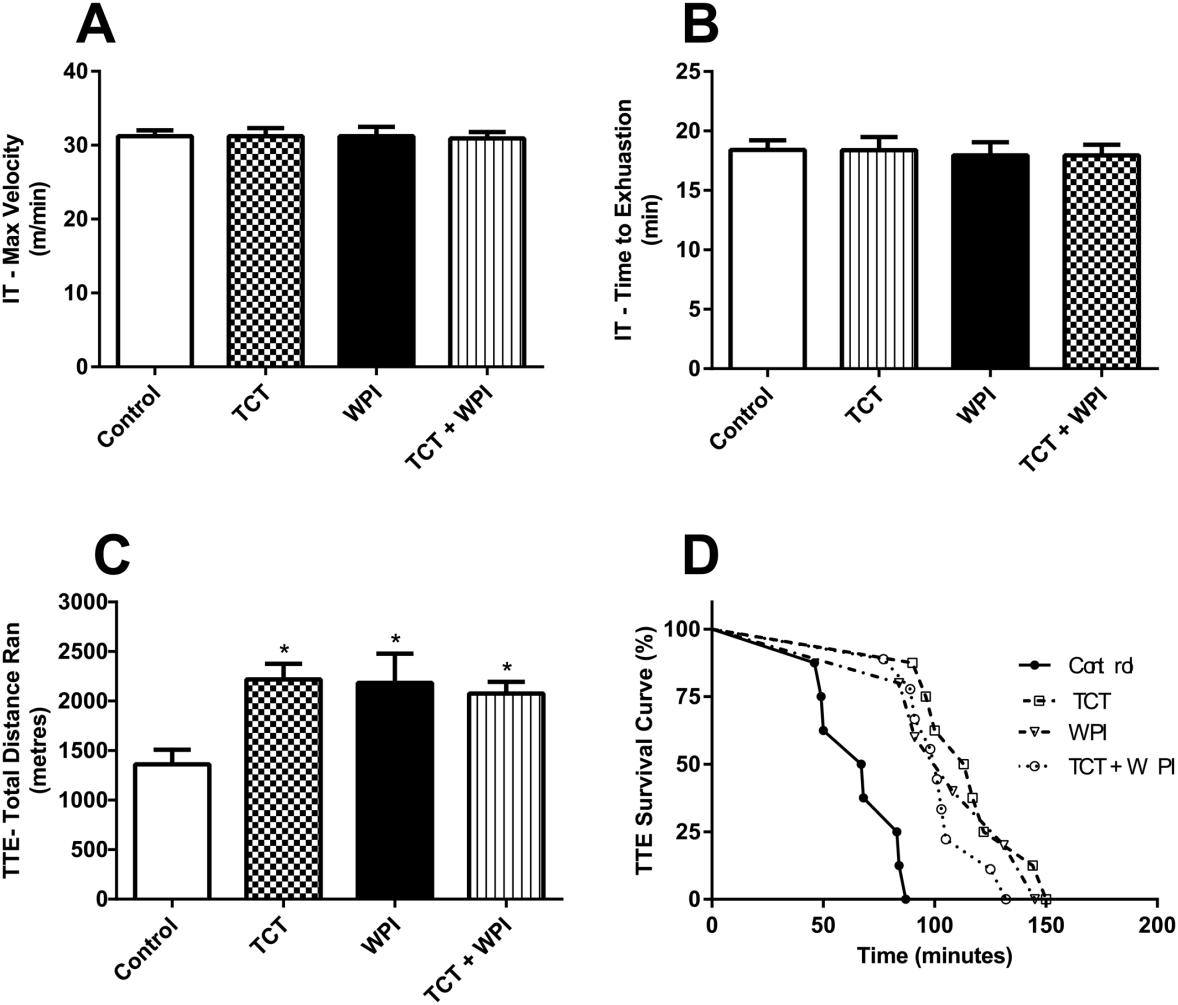
Maximal running velocity and running distance in the IT and the TTE respectively. Average peak velocity (A) and maximal running time (B) achieved for the IT performed at week 8. Treadmill speed was increased 2.5 m/min every 2 min starting from 10m/min until exhaustion. Average distance run during the TTE (C) and total run time (D) survival curve. Data shown as mean ± SEM (n = 5−9/group).* Signifies a statistically significant difference (P<0.05) compared to the Control group. There were no differences between the supplemented groups for any of these measures. Control = HFD alone; TCT = HFD+tocotrienol; WPI = HFD+whey protein isolate; TCT+WPI = HFD+tocotrienol+whey protein isolates; IT = Incremental Test; TTE = Test to Exhaustion.

During incremental testing (IT), there was no difference in the average peak speed reached (~31 m/min) and the total exercise time between the groups (Fig 4A). In contrast, when performing the TTE after 10 weeks of treatment, the TCT (2271 ± 185m), WPI (2195 ± 265m), and TCT+WPI (2067 ± 104m) groups ran significantly further than the Control group (1428 ± 139m) (*P*<0.05) (Fig 4C), with no difference between the supplemented groups. Likewise, the total running time was significantly higher for the TCT, WPI and TCT + WPI groups compared to the Control group, with no difference between the supplemented groups (Fig 4D).

### Post-Exercise Plasma Glucose and Lactate Concentrations

**Fig 5:**
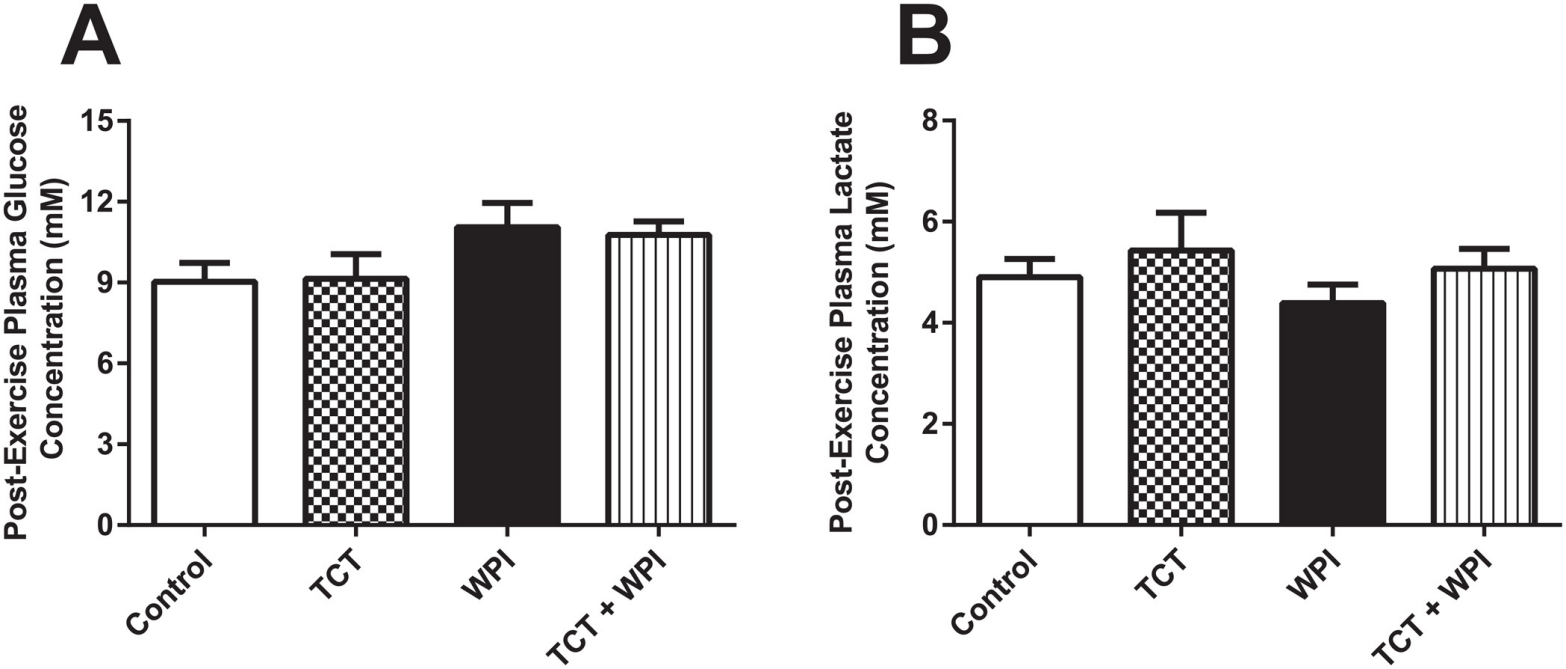
Plasma glucose (A) and lactate concentrations (B) following the TTE. Data shown as mean ± SEM (n = 5−9/group); no significant differences exist. Control = HFD alone; TCT = HFD+tocotrienol; WPI = HFD+whey protein isolate; TCT+WPI = HFD+tocotrienol+whey protein isolate.

Neither TCTs nor WPIs significantly influenced post-exercise plasma glucose levels compared with the Control group (Fig 5A), although a slight trend for WPI (p = 0.11) and WPI + TCT (p = 0.06) to be higher than Control, this did not reach statistical significance. Similarly, with respect to post-exercise plasma lactate levels, there was no difference between groups (Fig 5B).

### Skeletal Muscle Mitochondrial Enzyme Activity

**Fig 6:**
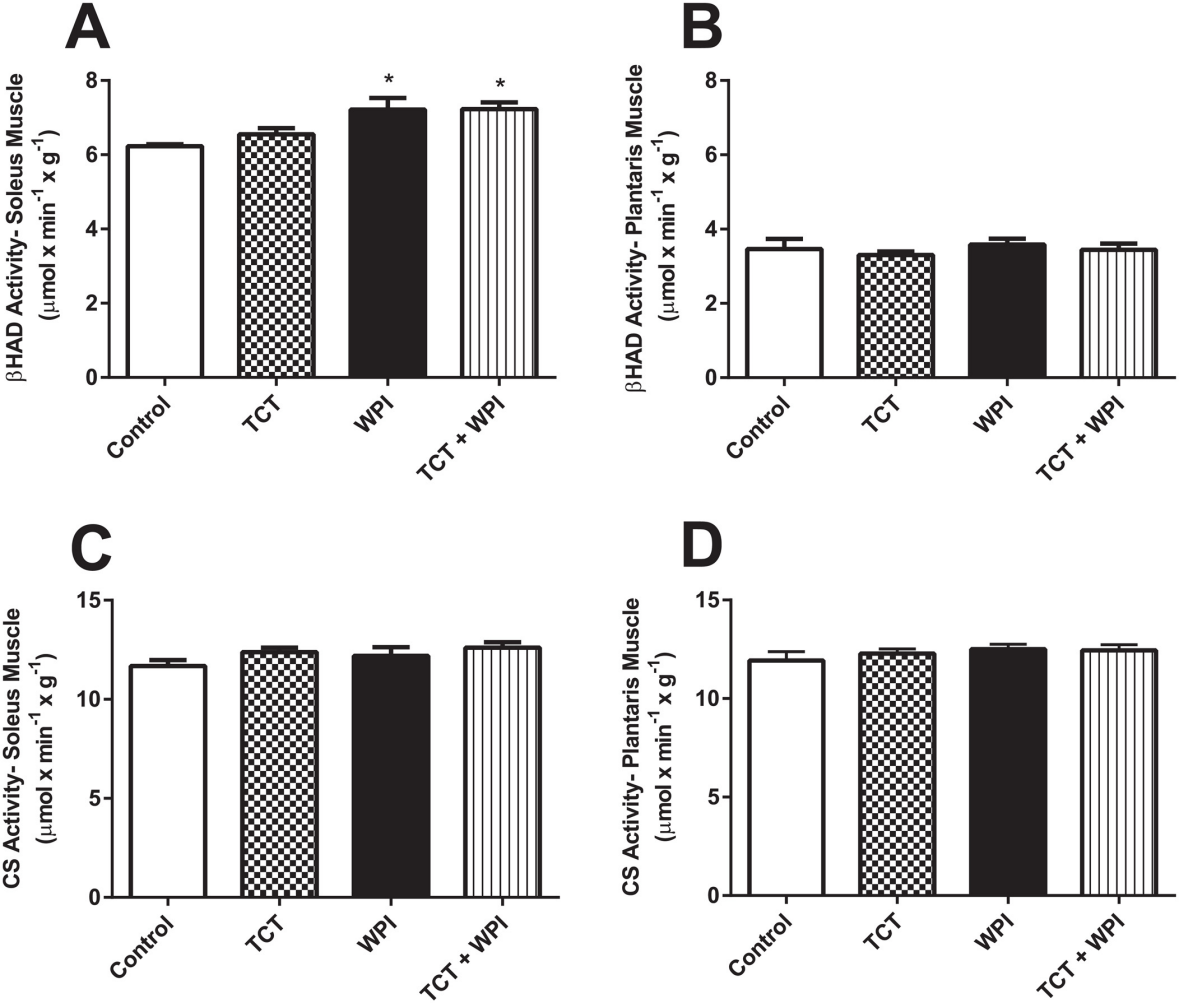
β-HAD activity in the soleus (A) and plantaris (B) muscles, and CS activity in the soleus (C) and plantaris (D) muscles. All data shown as mean ± SEM (n = 6−8/group). * denotes a statistical significant difference compared to the Control group P<0.05 Control = HFD alone; TCT = HFD+tocotrienol; WPI = HFD+whey protein isolate; TCT+WPI = HFD+tocotrienol+whey protein isolate; β-HAD = beta-hydroxyacyl-CoA; CS = citrate synthase.

The WPI and TCT + WPI groups had an approximately 16% higher (*P*<0.05) maximum *in vitro* β-HAD enzyme activity in the soleus muscle compared with the Control group (Fig 6A) with no significant difference in the plantaris muscle (Fig 6B). CS activity was not different between groups for both muscles (Fig 6C+6D).

### Skeletal Muscle and Liver Glycogen Content

**Fig 7:**
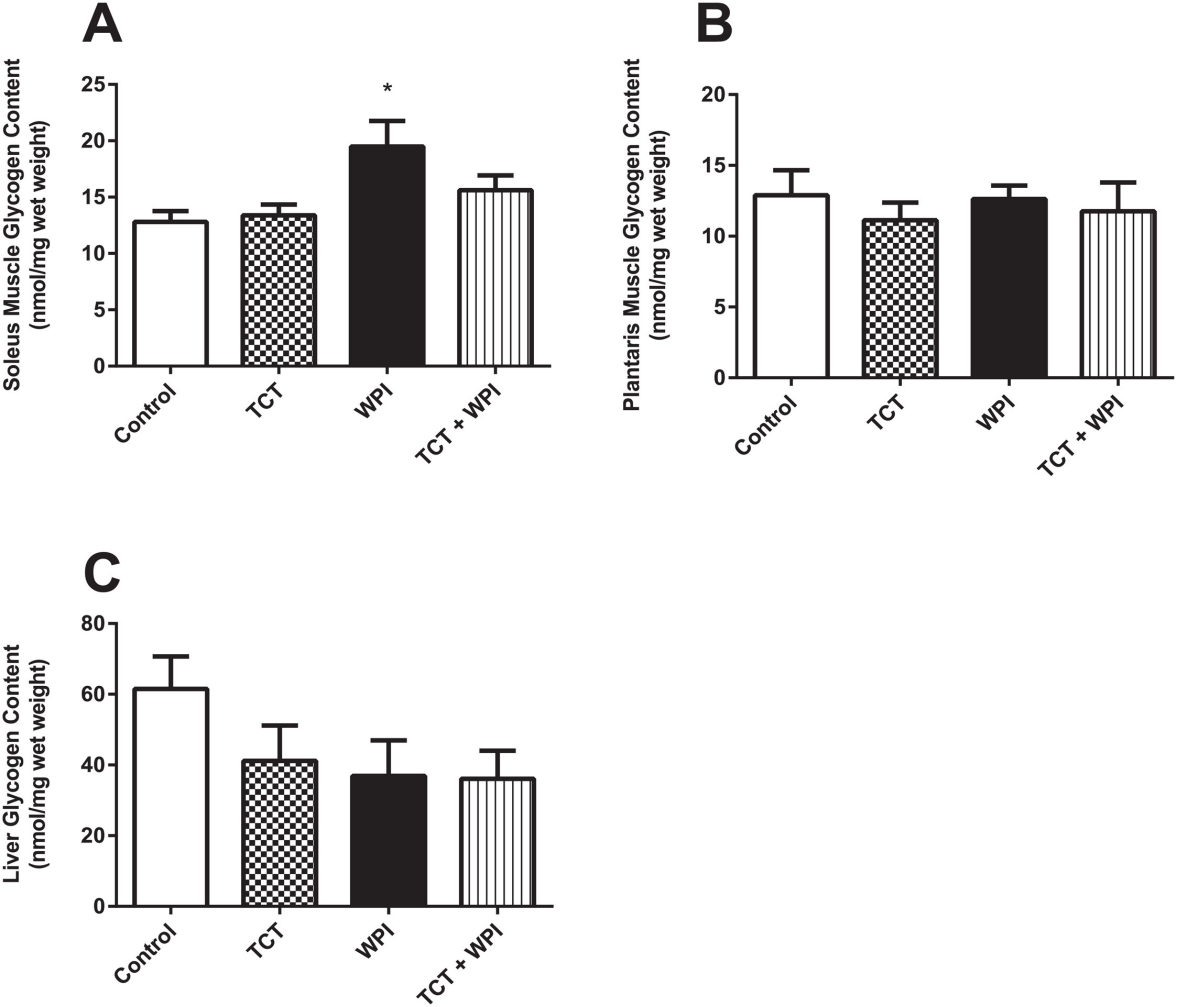
Post-exercise glycogen content in the soleus (A), plantaris (B) and the liver (C). All data shown as mean ± SEM (n = 5−8/group) and there were no significant differences (p>0.05). Control = HFD alone; TCT = HFD+tocotrienol; WPI = HFD+whey protein isolate; TCT+WPI = HFD+tocotrienol+whey protein isolate.

In the soleus, the WPI supplementation group had a significantly higher post-exercise glycogen content compared to the Control group (Fig 7A) and was not different compared to the other groups. There were no differences between any of the groups in glycogen content after exercise for the plantaris (Fig 7B) or the liver (Fig 7C).

## Discussion

This study demonstrated that tocotrienol and whey protein supplementation substantially improved endurance exercise capacity in HFD fed rats. To our knowledge this is the first study demonstrating that animals on a HFD supplemented with TCTs and/or WPIs show a remarkable increase in exercise capacity compared with HFD only animals. This novel finding provides new awareness of the potential of these naturally occurring nutrients to increase exercise tolerance in populations at-risk of developing obesity and potentially subsequent insulin-resistance, which in turn could aid in the amelioration or prevention of these diseases.

Our study demonstrates that sedentary male rats receiving a HFD supplemented with TCTs and/or WPIs for 10 weeks performed approximately 50% better during a submaximal running test to exhaustion compared to HFD only control group. One previous study, in lean rats, showed that 4 weeks of TCT supplementation improved swimming time to exhaustion by nearly 2-fold compared with controls [33]. Furthermore, despite swimming nearly 100 min longer, the TCT fed rats had nearly 40% more muscle and liver glycogen after exercise, suggesting a significant glycogen sparing effect [33]. We wanted to expand on this finding to determine if these results are translatable to diet-induced obese rats. In our study, supplemented animals ran 60% further and for a longer time than control animals, which is a remarkable difference, although lower than the 2-fold increase reported previously in lean rats [33]. Secondly, in contrast to Lee and colleagues [33], the TCT group did not have higher post-exercise muscle or liver glycogen content.

It is difficult to reconcile the difference in exercise time and glycogen levels between the two studies, although several differences in these studies could explain the discrepancy. Firstly, Lee and colleagues [33] used swimming exercise which may cause a different stress and physiological response [49] compared to running which is a normal and natural activity for rats. Secondly, it is difficult to know if the relative intensity of the swimming exercise and running exercise are comparable, and it is likely that differences in the relative intensity of exercise will affect substrate usage and perhaps the effects of TCT. It is important to note that all groups achieved the same maximal running velocity in the incremental running test and all had the same body mass and adiposity which are important factors that could alter the relative intensity for weight bearing exercise such as running. As such, we are confident that all groups were exercising at the same relative intensity, which is also confirmed by similar post-exercise plasma lactate concentrations. Thirdly, we used a diet-induced obese model compared to Lee et al. (2009) who used normal chow-fed lean rats. All three of these factors can affect the selection of metabolic substrates during exercise, and may also interact to cause an altered response during exercise. Thus it is difficult to directly compare the two studies. However, the substantial benefit of TCTs to increase submaximal intensity exercise endurance is consistent between these two studies despite in our model the presence of 10 weeks of high fat feeding to elicit diet-induced obesity [37, 38].

One of the purported benefits of TCTs is a potential increase in fatty acid oxidation suggested to be due to increase in PPAR activity and subsequent mitochondrial biogenesis [31, 32]. Consistent with this, the study by Lee and colleagues [33] showing increased exercise endurance, glycogen sparing and lower lactate levels following exercise suggests a greater reliance on fatty acids throughout the exercise. With the exception of the WPI group having higher glycogen for the soleus only, all groups in the current study had the same glycogen and the same plasma glucose and lactate concentrations following exercise, suggesting that carbohydrate oxidation, and by extension fat oxidation, was the same across the groups. If the TCT group did preferentially use fatty acids over carbohydrates, then we would expect lower plasma lactate and higher muscle glycogen after the exercise. It may be speculated that the same muscle glycogen content at the end of exercise despite longer exercise durations represents a glycogen sparing effect of the supplements. However, caution in this interpretation is necessary since we do not know the pre-exercise muscle glycogen levels, and thus it is possible that the supplementation may have resulted in a higher (or lower) glycogen content. One study comparing whey protein with casein protein supplementation found that whey protein increases skeletal muscle and liver glycogen content [50] compared to casein protein, however since they did not have a control group we cannot determine if whey supplementation is more beneficial or if casein is detrimental compared to a normal diet, and also cannot infer if a HFD alters this observed difference in whey versus casein on glycogen levels. We did observe that the WPI group had higher post-exercise glycogen in the soleus, but not in the plantaris, but without knowing the pre-exercise glycogen levels, it is difficult to interpret this data conclusively.

There is some evidence that whey protein, or its components, can increase fatty acid oxidation by increasing mitochondrial biogenesis [24, 51]. We used CS activity as a marker for mitochondrial content [40, 52, 53], to assess if supplementation of TCT or WPI increased mitochondria and thus the capacity to oxidize fatty acids. In both the soleus and plantaris there was no difference in CS activity amongst the groups and therefore there was no evidence of enhanced mitochondrial content with the treatments. This fits with the IT results in which all groups achieved the same peak velocity and presumably maximal aerobic capacity [40, 41], although maximal aerobic capacity may be more a determinant of central limitations rather than peripheral limitations. We also measured maximal activity of β-HAD *in vitro* to determine if there was a greater inherent capacity to oxidize fatty acids and found that WPI’s increased β-HAD maximal activity in the soleus, but not in the plantaris. Both the WPI and the TCT + WPI groups displayed this increase. It is possible that WPIs increase the capacity of skeletal muscle to utilize fatty acids and thus preserve carbohydrate stores, allowing for greater exercise duration. Indeed, the soleus had higher post-exercise glycogen content in the WPI group, suggesting increased fatty acid utilization and some glycogen sparing. However, this does not explain the improved exercise capacity of the TCT group, as the activity of β-HAD and CS were similar to the control group for both muscles. Recently it has been shown that long-term TCTs supplementation increases mitochondrial membrane potential and ATP concentrations in the brains of mice [54], which is also supported by protective effects against mitochondrial dysfunction in liver cells in culture [55]. If mitochondrial function were improved by TCTs, then this could explain the significantly improved performance in the TTE for the TCTs group. Perhaps this benefit on the mitochondria is only manifested during exercise of longer duration, which explains the benefit of TCTs during the TTE and not during the shorter IT for which cessation of exercise is likely from a different cause.

The results from this current investigation suggesting that WPIs increase the capacity for skeletal muscle to oxidise lipids are supported by several other published findings. Zhou and colleagues [51] reported that rodents on a high WPI-based diet (24% WPI) for 12 weeks exhibited reduced 48 h respiratory quotient indicating that WPI increased whole-body fatty acid oxidation. More recently, Hill and colleagues [20] reported that 2 weeks of WPI-supplementation (approximately 9% of total energy intake- similar to the amount used in this current investigation) significantly increased PGC-1α gene expression 6 hours after an exercise bout in the skeletal muscle of trained cyclists. Meanwhile, whey protein supplementation in combination with exercise training improved exercise performance more than whey protein or exercise training alone, although there was not a significant difference between whey protein and control diet groups [56].

It has been suggested that high-fat diets may disturb normal mitochondrial function which contributes to metabolic disturbances, with some evidence suggesting TCT may serve a protective role in obesity [31]. One of the questions of this study was to determine if TCT could attenuate diet-induced changes in body weight and adiposity; however in the current study there was no evidence of a protective effect of TCT on diet-induced obesity. All groups had similar body weight and adiposity and there was also no difference in IPGTT or IPITT between the Control group and the supplemented groups. Some of the purported benefits of TCTs are an activation of PPARs which can then lead to increased enzymes involved in mitochondrial respiration and fatty acid oxidation. While we did not measure these proteins directly, based on the lack of difference in the TCT group compared to the Control group in β-HAD activity, CS activity, adiposity, end of exercise glycogen levels or plasma lactate, there is little evidence of an enhanced fatty acid oxidation or mitochondrial content in the TCT group.

While the benefit of these supplements on submaximal exercise capacity is significant, some limitations in this study warrant comment and should be considered for future studies. Whilst this study controlled the relative exercise intensity of all rats performing the final TTE, the use of indirect calorimetry to measure oxygen uptake and respiratory exchange ratio or radioactive tracers would have given more powerful insight into the proportion of fat or carbohydrate oxidation in all groups. Although it is likely that these animals would have displayed a metabolic disease phenotype since only 4 weeks of HFD has been shown to induce insulin resistance [57], the current study would have benefited by including a lean control group. Furthermore, the more rigorous hyperinsulinemic euglycemic clamp technique [58] along with possible tracer methodology would have offered greater accuracy in determining the effects of TCTs and WPIs on glucose homeostasis and insulin resistance in these diet-induced obese rats. Lastly, this study did not measure any markers of oxidative stress or endogenous/exogenous antioxidant systems, primarily because all tissues were collected after an intense exercise bout, and thus these markers would not reflect the basal state or any changes due to the 10 weeks of diet supplementation.

## Conclusion

In conclusion, 10-weeks of TCT- and/or WPI-supplementation improved exercise capacity in male sedentary Sprague-Dawley rats fed a HFD compared with non-supplemented HFD fed rats. In terms of WPIs, we postulate this observed effect to be attributed, at least in part, to the observed increase in β-HAD enzyme activity in the soleus muscle of the rats which may have improved endurance by increasing fat utilisation and sparing muscle glycogen. The mechanisms pertaining to the effect of TCTs on exercise endurance in this study are unclear but may relate to increased mitochondrial membrane potential [54]. This study provides rationale for future investigative studies to explore the mechanistic pathways involved in the endurance exercise-enhancing effects mediated by WPIs and TCTs in diet-induced obese rats. Future investigations into the oxygen consumption, via indirect calorimetry, and use of tracer technology to determine glucose utilisation and fatty acid oxidation in models of diet-induced obesity are also warranted. Furthermore, it will be essential to follow up using a female cohort to show that the benefits of the supplementations are generalizable. Given that exercise tolerance is appreciably lower in obese/diabetic cohorts, these findings point to a potential use of these nutrients as a possible treatment modality.

## Abbreviations

β-HAD: Beta-hydroxyacyl-CoA
CS: Citrate synthase
HFD: High fat diet
IPGTT: Intraperitoneal glucose tolerance test
IPITT: Intraperitoneal insulin tolerance test
IT: Incremental exercise test
PGC-1α: peroxisome proliferator-activated receptor gamma coactivator 1 alpha
SOL: Soleus
TCT: Tocotrienol
TTE: Test to Exhaustion
WPI: Whey protein isolates
